# Incidence of imipenem-resistant Acinetobacter baumannii in a general intensive care unit (ICU)

**Published:** 2014

**Authors:** Mostafa Alavi-Moghadam, Mirmohammad Miri, Majid Mokhtari, Mehran Kouchek, Reza Goharani, Mohammad Sistanzad, Saeed Safari, Mehrdad Solouki

**Affiliations:** 1 Department of Emergency Medicine, Emam Hossein Medical and Educational Center, Shahid Beheshti University of Medical Sciences, Tehran, Iran; 2 Department of Critical Care Medicine, Emam Hossein Medical and Educational Center, Shahid Beheshti University of Medical Sciences, Tehran, Iran; 3 Department of Clinical Pharmacy, Shahid Beheshti University of Medical Sciences, Tehran, Iran.; 4 Department of Internal and Critical Care Medicine, Emam Hossein Medical and Educational Center, Shahid Beheshti University of Medical Sciences, Tehran, Iran

**Sir**

Hospital-acquired infections in critically ill patients remain one of the most important contributors to morbidity and mortality. Hospital-acquired A. baumannii has emerged as a serious threat to ICU patient population. The main purpose of our study was to determine the incidence of ICU acquired imipenem-resistant A. baumannii and its impact on ICU mortality.

This prospective study was carried from January 2010 to October 2010 in a general ICU of a major teaching hospital in Tehran, Iran. The patients who had positive culture of A. baumannii were included in the study. A. baumanni isolates were tested for the sensitivity or resistance to imipenem by E-test. Each test contains 10 mcg of imipenem. Sensitivity to imipenem was defined as less than 4 mcg/ml. Sixty one positive cultures from 61 patients were detected during the study period. The most frequent infection was ventilator-associated pneumonia. Fifty (82.5 %) of isolates were detected in sputum, 5 (8%) in wounds, 3 (4.9%) in urine, 2 (3%) from central venous line and 1 (1.6) in blood. All isolates were imipenem resistant. The resistance level determined by the E-test was 12 mcg/ml in 1% of patients, 16 in 4%, 24 in 5%, and 32 in 88% of isolates. In this study, A. baumannii displayed 100% resistance to imipenem. Other study has reported imipenem resistance in the range of 11-24% (1). In a report from 5 European countries, Acinetobacter species were found to have the highest increase in resistance to antibiotics among all the gram-negative bacilli studied (2). Several risk factors have been identified for developing infection with multidrug- resistant A. baumannii, including admission diagnosis of multiple trauma, mechanical ventilation, length of ICU stay, colonization pressure within the unit and exposure to antimicrobials agents (3- 6).

The alarming imipenem resistance in this study can be due to inappropriate choice of antibiotics and doses. In one large multicenter study in US hospitals, acinetobacter blood stream infection was associated with a crude mortality rate of 34% (7). The reviewed data suggest that infection with or the acquisition of A. baumannii seems to be associated with increased mortality (8). 

A.baumannii has been found on inanimate surfaces even after cleaning with antiseptic solutions (9). Healthcare workers play a potential role in epidemic outbreaks through the contamination of the environment and other patients.

However, this very alarming study conveys important messages that strict infection control measures have to be put in place by infection control department. In summary, considering the unacceptably of the high incidence of imipenem resistant A. baumannii infections with its significant morbidity, mortality and costs, it is mandatory to improve infection control strategies and optimize its diagnosis and management.
